# Influence of endodontic motors on the behaviour of root canal shaping instruments: an in vitro comparative study

**DOI:** 10.1038/s41405-023-00179-z

**Published:** 2023-11-29

**Authors:** Clémentine Reynette, Renaud Giess, Jeanne Davril, Jean-Marc Martrette, Éric Mortier, Rémy Balthazard, Marin Vincent

**Affiliations:** 1 Faculté d’odontologie de Lorraine, Université de Lorraine, Nancy, France; 2 Départment de Dentisterie Restauratrice et Endodontie, Faculté d’odontologie de Lorraine, Université de Lorraine, Nancy, France; 3 CNRS, IJL, Université de Lorraine, Nancy, France; 4 Université de Lorraine, Faculté de Médecine, EA 3450, Développement, Adaptation et Handicap, Nancy, France; 5 CNRS, LEM3, Université de Lorraine, Metz, France

**Keywords:** Dental equipment, Endodontics

## Abstract

**Aims:**

The endodontic literature reports a lot of comparative study on endodontic instruments, concerning as well their geometry, instrumental dynamics, material, mechanical behavior or heat treatment. However, to our knowledge, no study has focused on the influence of endodontic motors on the shaping abilities of endodontic instruments. Thus, the aim of this study was to analyze the influence of the endodontic motors on root canal shaping instruments.

**Method:**

Dual Move (MICRO-MEGA, Besançon, France), Canal Pro CL2i (COLTENE, Alstätten, Suisse), Canal Pro Jeni Motor (COLTENE, Alstätten, Suisse), Ai Motor (WOODPECKER, Guilin, China), Wave One motor (VDW, Postfach, Munich) and Smart A (WOODPECKER, Guilin, China) were pre-clinically compared in continuous rotation and reciprocating motion on a traction/compression bench using resin blocks. Canal shaping in continuous rotation and reciprocating motion were performed with One Curve and One RECI instruments (MICRO-MEGA, Besançon, France), respectively. The penetration/removal forces, making it possible to objectify the cutting effect and screwing effect of the instruments during root canal shaping, were analyzed.

**Results:**

The results showed (i) that endodontic motors influence the mechanical behavior of endodontic instruments, (ii) that the influence of the motors is essentially felt during reciprocating motion and (iii) that the reciprocating angles influence the mechanical behavior of endodontic instruments.

**Conclusion:**

Only endodontic instruments are widely studied in literature while endodontic motors have a direct influence on root canal treatment. This study analyzes the influence of the endodontic motors on root canal shaping instruments. This study tends to demonstrate that Jeni Motor could optimize the mechanical behavior of endodontic instruments.

## Introduction

Since the advent of nickel titanium and the democratization of mechanized endodontic instruments, the endodontic market has continuously developed to offer safer and more efficient solutions [1–3]. In this context, instrumental developments in terms of materials [4–6], geometries [7–12], kinematics [13–22], surface [5, 23–27] and thermal treatments [28–33] have gradually allowed to offer more reproducible endodontic treatments. By taking these different parameters one by one as variable, several mechanical and pre-clinical studies have made it possible to compare these instruments in terms of flexibility [10, 28, 34–46], cyclic fatigue [19, 36–38, 42, 47–57], resistance to torsion [43–45, 58, 59] and separation incidence. [10, 60–64] The aim of these endodontics developments is to limit therapeutic errors as much as possible, especially instrument breakage [65].

In parallel with these instrumental developments, endodontic motors and kinematics have also been widely developed [66–70]. Historically on the dental unit, these motors had only one mode of continuous rotation without any torque control. Then came the first dedicated endodontic motors with torque control, then the first non-wired motors. Subsequently, different instrumental dynamics were implemented, among which we can cite the reciprocating motion. More recently, manufacturers have offered “intelligent endodontic motors”, as such as the Canal Pro Jeni (COLTENE, Alstätten, Germany), the TriAuto ZX2+ (MORITA, Osaka, Japan) or the EndoPilot (KOMET, Lemgo, Germany), allowing to adapt its movement by analyzing the torsion constraints in real time.

Today, all endodontic manufacturers propose endodontic motors in addition to their range of instruments. However, the scientific literature has not extensively investigated endodontic motors and the studies generally highlight the non-reproducibility between kinematics settings and manufacturers’ declared values [70–74], torque analysis [68, 75–78] or integrated apex locator [79–84]. To our knowledge, no study has been properly carried out on the impact of the endodontic motor on root canal shaping. Similarly, no study has made it possible to highlight an equivalence or not between these motors, their different modes or instrumental dynamics.

Therefore, by taking endodontic instruments as a fixed parameter, this paper proposes to study endodontic motors as a variable by pre-clinically comparing them by penetration/removal preclinical tests.

## Materials and Methods

### Fixed parameters

#### Resin blocks

120 resin blocks (DENTSPLY SIRONA, Ballaigues, Switzerland) were used for the performance and security tests on traction / compression bench.

**Fig. 1:**
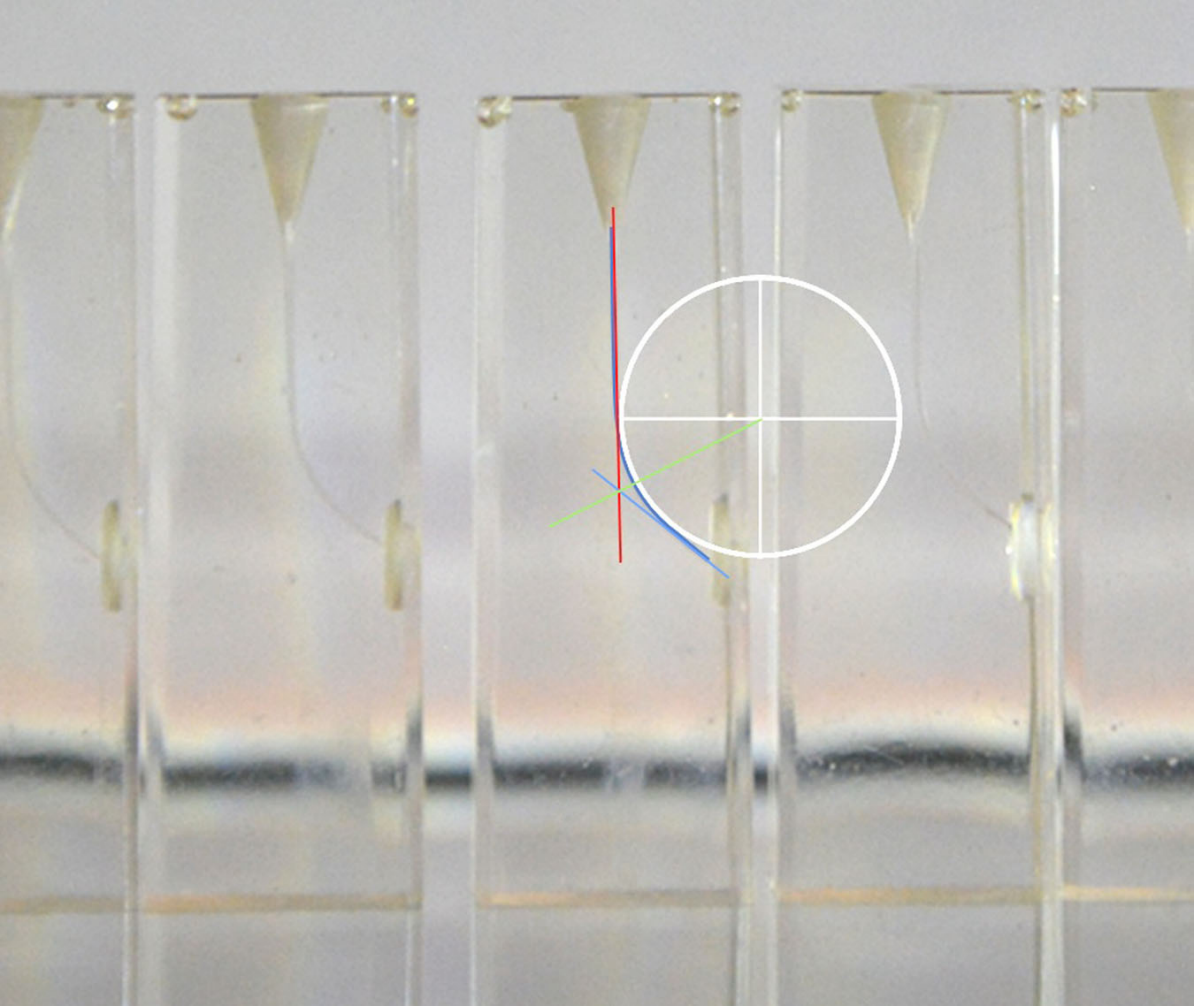
DENTPLY MAILLEFER endodontic resin block. The blue and red lines are the tangents of the white circle. The intersection of the green line and the blue curve marks the point where the bending is maximum. The radius of the white circle represents the radius of curvature.

The DENTSPLY SIRONA resin blocks have an average length of 18 mm with an apical permeability, an average radius of curvature of 4.5 to 5 mm and an average curvature of 30 to 50° (Fig. 1). According to the AAE Endodontic Case Difficulty Assessment Form and Guidelines, these endodontic blocks simulate complex endodontic cases.

For each tested endodontic motor, 10 blocks were shaped in continuous rotation and 10 blocks in reciprocating motion.

#### Root canal shaping instruments

Concerning the continuous rotation tests, all the resin blocks were shaped using the following protocol:

- permeabilization using a K.10 file (MICRO-MEGA, Besançon, France);
- shaping using the One Curve instrument (MICRO-MEGA, Besançon, France).
  Concerning the reciprocating motion, all the resin blocks were shaped using the following protocol:
- permeabilization using a K.10 file (MICRO-MEGA, Besançon, France);
- shaping using the One RECI instrument (MICRO-MEGA, Besançon, France).

### Variable parameters

The tested endodontic motors are DualMove (MICRO-MEGA, Besançon, France), Canal Pro CL2i (COLTENE, Langenau, Germany), Jeni Motor (COLTENE, Langenau, Germany), Ai Motor (WOODPECKER, Guilin, China), Wave One motor (VDW, Postfach, Munich) and Smart A (WOODPECKER, Guilin, China).

For the continuous rotation tests, all the motors were set at a speed of 350 rpm and a maximum torque of 2.5 N.cm, in accordance with the One Curve manufacturer recommendations. Contrary to the other motors, the Jeni Motor presents a specific program for One Curve with an assistance system that control the variable instrument movements.

For the reciprocating motion tests, all the motors could not be configured according to the One RECI manufacturer recommendations due to non-present reciprocating angle ranges. In addition, all motors could not associate a speed or a maximum torque during reciprocating motion mode. The reciprocity parameters were:

- Dual Move: 60 °CW/170 °CCW (the manufacturer does not offer the possibility to change rotation speed and maximum torque);
- Canal Pro CL2i: 30 °CW/150 °CCW, rotation speed 350 rpm and maximum torque 4 N.cm (the manufacturer does not offer the possibility to set the One RECI angle parameters);
- Jeni Motor: equivalent of 60 °CW/170 °CCW set in milliseconds, rotation speed 350 rpm and maximum torque 4 N.cm (the Jeni Motor doesn’t present a specific assistance system for One Reci; it was used with the “Doctor’s choice” mode);
- Ai Motor: 60 °CW/170 °CCW, rotation speed 350 rpm and maximum torque 4 N.cm;
- Wave One Motor: 30 °CW/150 °CCW (the manufacturer does not offer the possibility to change rotation speed, maximum torque none to set the One RECI angle parameters);
- Smart A: 60 °CW/170 °CCW, rotation speed 400 rpm, maximum torque 4 N.cm (the manufacturer offers a non-editable One RECI program with a 400 rpm rotation speed).

### Penetration/removal preclinical tests

The development of an endodontic penetration/removal (P/R) protocol was carried out on a traction machine (Zwick/Roell-50 N force cell, Ulm, Germany) controlled by the testXpert II software (Zwick/Roell) in the LEM3 laboratory (PolyTech, Nancy, France) [85]. This protocol corresponds to a free P/R test involving 25 successive charge/discharge cycles, divided into 9 groups of cycles, allowing the descent and the work of the tested endodontic instrument in a resin block having a radius of curvature about 4.5 mm (DENTSPLY SIRONA, Ballaigues, Switzerland). Complete instrumental removal is performed between each cycle allowing (i) a canal irrigation to remove debris and (ii) a verification of the maintenance of apical patency. The vertical components of the force and displacement are measured. At the end of each test, the maximum penetration and removal forces are recorded for each group of cycles by testXpert II software (Zwick/Roell). Only the max torque was defined. No real-time measures of torque has been made. This protocol was established in order to respect the clinical reality and to be reproducible. It shows three groups of parameters appropriate to the work of the associated canal section:

- Canal penetration from 10 to 14 mm, before the curvature (Table 1),

**Table 1:** P/R protocol in continuous rotation: penetration from 10 to 14 mm.

			Penetration [mm]	Speed [mm/s]	Stop [s]
Group 1	Cycle 1	Charge	11	5	
		Discharge	10.5	5	
	Cycle 2	Charge	11.5	5	
		Discharge	11	5	
	Cycle 3	Charge	12	5	
		Discharge	11.5	5	
	Irrigation		−51	15	5
Group 2	Cycle 4	Charge	12.5	5	
		Discharge	12	5	
	Cycle 5	Charge	13	5	
		Discharge	12.5	5	
	Cycle 6	Charge	13.5	5	
		Discharge	13	5	
	Irrigation		−51	15	5
Group 3	Cycle 7	Charge	14	5	
		Discharge	13.5	5	
	Cycle 8	Charge	14.5	5	
		Discharge	14	5	
	Cycle 9	Charge	15	5	
		Discharge	14.5	5	
	Irrigation		−51	15	5

The protocol considers the 1 mm wedge positioned between the instrument and the resin block.
- Canal penetration from 14 to 16 mm, in the most coronal part of the curvature (Table 2),

**Table 2:** P/R protocol in continuous rotation: penetration from 14 to 16 mm.

			Penetration [mm]	Speed [mm/s]	Stop [s]
Group 4	Cycle 10	Charge	15.25	2	
		Discharge	15	2	
	Cycle 11	Charge	15.5	2	
		Discharge	15.25	2	
	Cycle 12	Charge	15.75	2	
		Discharge	15.5	2	
	Cycle 13	Charge	16	2	
		Discharge	15.75	2	
	Irrigation		−51	15	5
Group 5	Cycle 14	Charge	16.25	2	
		Discharge	16	2	
	Cycle 15	Charge	16.5	2	
		Discharge	16.25	2	
	Cycle 16	Charge	16.75	2	
		Discharge	16.5	2	
	Cycle 17	Charge	17	2	
		Discharge	16.75	2	
	Irrigation		−51	15	5

The protocol considers the 1 mm wedge positioned between the instrument and the resin block.
- Canal penetration from 16 to 18 mm, in the most apical part of the curvature (Table 3).

**Table 3:** P/R protocol in continuous rotation: penetration from 16 to 18 mm.

			Penetration [mm]	Speed [mm/s]	Stop [s]
Group 6	Cycle 18	Charge	17.25	2	
		Discharge	17	2	
	Cycle 19	Charge	17,5	2	
		Discharge	17.25	2	
	Irrigation		−51	15	5
Group 7	Cycle 20	Charge	17.75	2	
		Discharge	17.5	2	
	Cycle 21	Charge	18	2	
		Discharge	17.75	2	
	Irrigation		−51	15	5
Group 8	Cycle 22	Charge	18.25	2	
		Discharge	18	2	
	Cycle 23	Charge	18.5	2	
		Discharge	18.25	2	
	Irrigation		−51	15	5
Group 9	Cycle 24	Charge	18.75	2	
		Discharge	18,5	2	
	Cycle 25	Charge	19	2	
		Discharge	18.75	2	
	Irrigation		−51	15	5

The protocol considers the 1 mm wedge positioned between the instrument and the resin block.

The endodontic instrument is mounted on an endodontic contra-angle connected to the programmable endodontic motor tested. The instrument under test is positioned to 1 mm from the occlusal edge of the resin block using a wedge.

The irrigation protocol was not standardized. Between each group of cycles, a first irrigation was made by the same operator with water until complete elimination of the coronal two-thirds debris. Then, the apical patency was verified with a K.10 file. Finally, a last irrigation was made to eliminate the debris from the apical third placed in suspension after the apical patency verification.

**Fig. 2:**
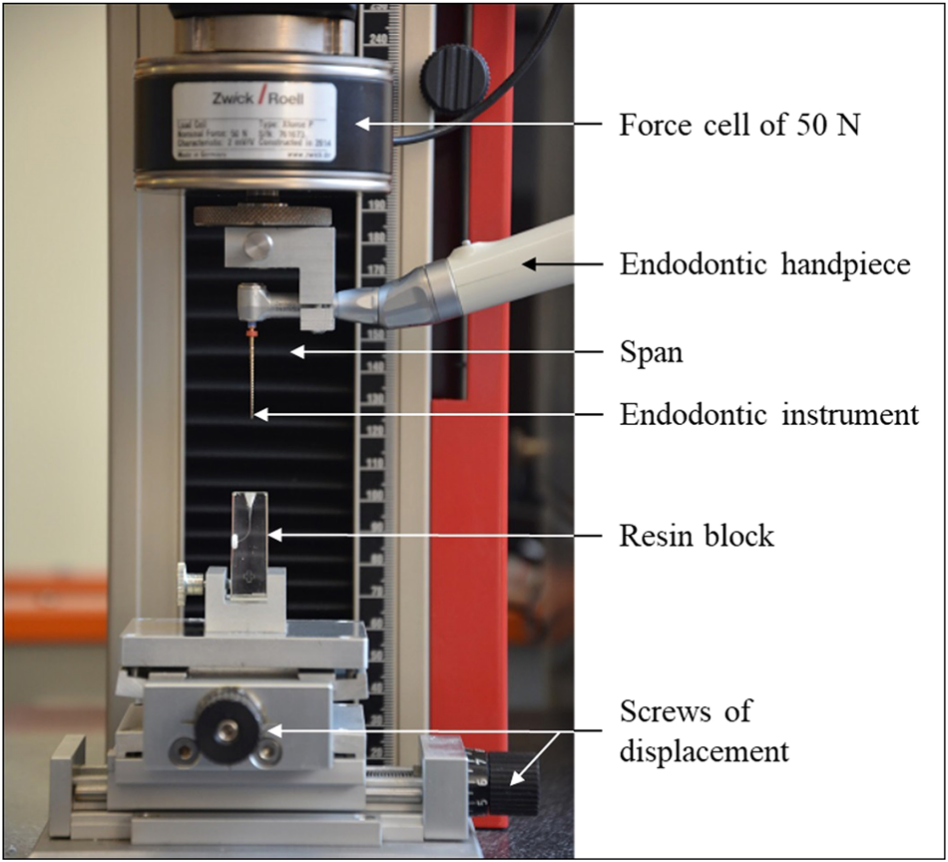
P/R bench used for this study. The black arrows show the different elements of the bench.

The penetration/removal bench is shown in Fig. 2.

### Statistics

Numerical data were analyzed using non-parametric Kruskall-Wallis-type statistical tests. A Dunns correction with α = 0.05 was performed for the two-by-two multiple comparisons. The results were considered statistically significant for a *P* value < 0.05. All statistical analyzes were performed using GraphPad Prism® 6 software (San Diego, California, US).

## Results

In our work, two types of forces are recorded by the force cell.

-Positive compressive forces which correspond to the penetration forces. Low penetration forces mean an effective cutting effect of the instruments.

-Negative traction forces which correspond to the removal forces. High removal forces mean high screwing forces driving the instruments in the apical direction.

These two types of forces are directly linked to the profile of the endodontic instrument. Therefore, instrument with cutting efficiency will tend to have higher screwing sensations.

### Continuous rotation

During continuous rotation kinetics, the influence of the endodontic motors on the mechanical behaviour of the instruments is low. Few significant differences are found for the Jeni Motor and the Dual Move. Concerning the Jeni Motor, the differences appear during all the steps of canal shaping. However, Dual Move differences only appear on the apical part during removal steps.

**Fig. 3:**
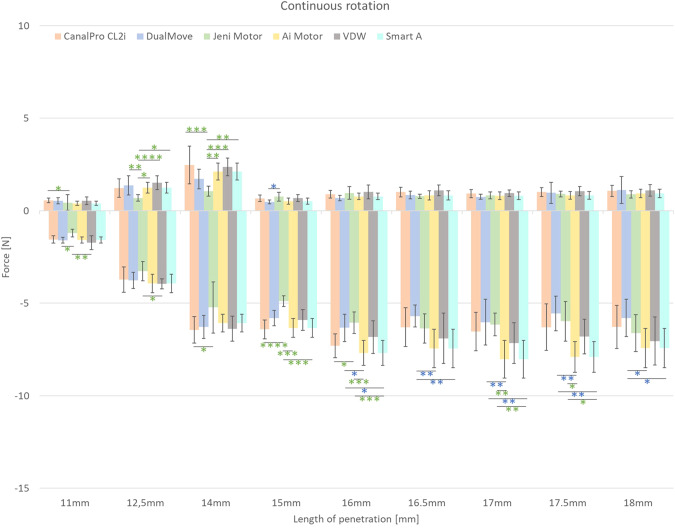
P/R results after shaping of the resin blocks with One Curve instruments drove by different endodontic motors (*P > 0.05; **P > 0.01; ***P > 0.001; ****P > 0.0001). The positive and negative forces represent the penetration and removal forces, respectively. When statistical significance is present, the statistical significance marks have the color of the best endodontic motor.

All the results in continuous rotation are reported in the Fig. 3 and Tables 4, 5.

**Table 4:** Statistically significant differences obtained in penetration during continuous rotation tests (*P < 0.05; **P < 0.01; ***P < 0.001; ****P < 0.0001).

Continuous Rotation	Dual Move	Canal Pro CL2i	Jeni Motor	Ai Motor	Wave One motor	Smart A
Dual Move		15 mm*				
Jeni Motor	12.5 mm**	11 mm* 14 mm***		12.5 mm* 14 mm**	12.5 mm**** 14 mm***	12.5 mm* 14 mm**

**Table 5:** Statistically significant differences obtained in removal during continuous rotation tests (*P < 0.05; **P < 0.01; ***P < 0.001; ****P < 0.0001).

Reciprocating Motion	Dual Move	Canal Pro CL2i	Jeni Motor	Ai Motor	Wave One motor	Smart A
Dual Move				16 mm* 16.5 mm** 17 mm** 17.5 mm** 18 mm*		16 mm* 16.5 mm** 17 mm** 17.5 mm** 18 mm*
Jeni Motor	11 mm*	14 mm* 15 mm**** 16 mm*		15 mm*** 16 mm*** 17 mm** 17.5 mm*	11 mm** 12.5 mm*	15 mm*** 16 mm*** 17 mm** 17.5 mm*

### Reciprocating motion

During reciprocating motion, several significant differences are found bringing to light the complexity of this kinetic. Its main findings that the Canal Pro CL2i motor had the worst influence on the mechanical behaviour of the instruments. Wave One Motor, Ai Motor and Jeni Motor seems better respecting the reciprocating requirements.

**Fig. 4:**
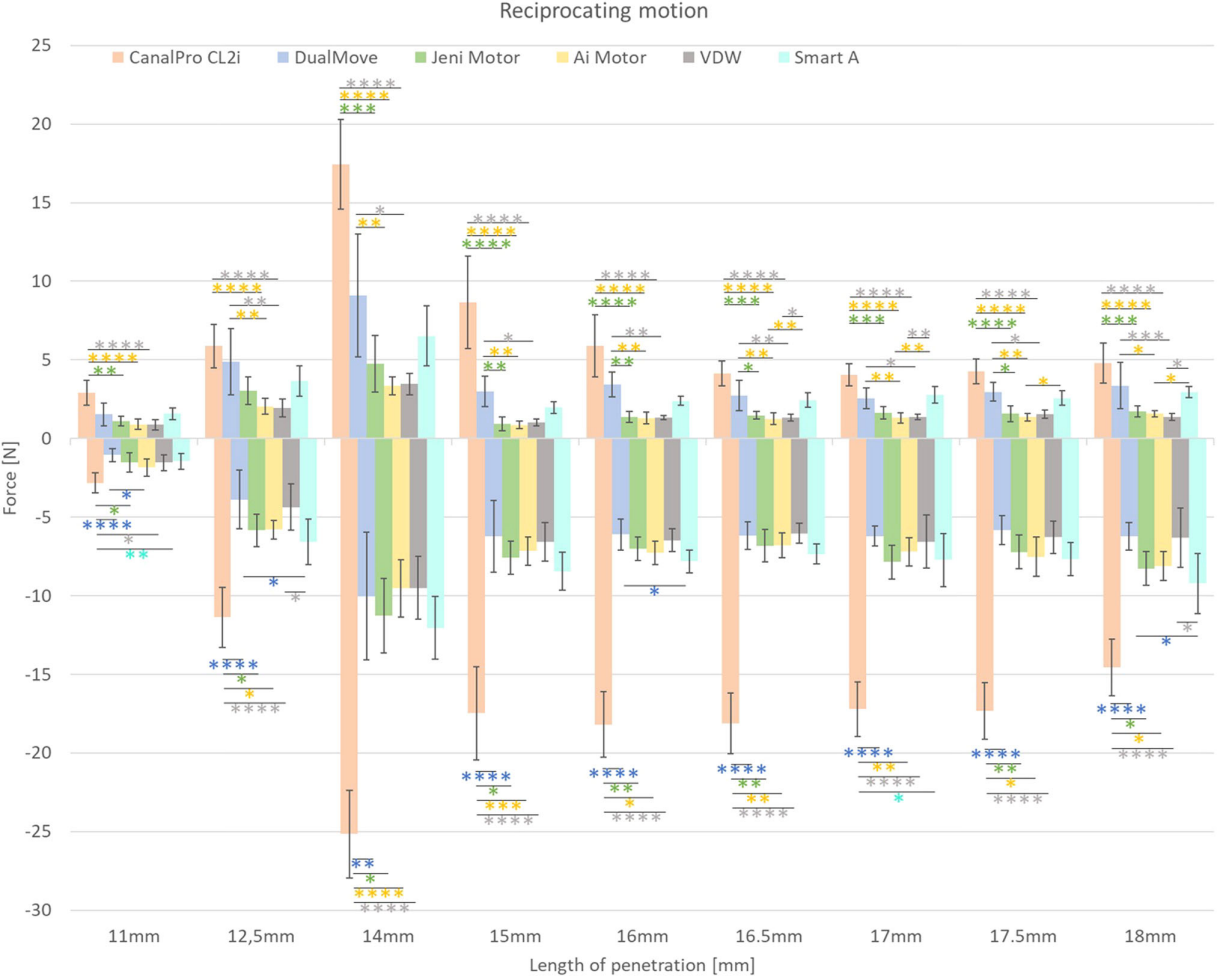
P/R results after shaping of the resin blocks with One RECI instruments drove by different endodontic motors (*P > 0.05; **P > 0.01; ***P > 0.001; ****P > 0.0001). The positive and negative forces represent the penetration and removal forces, respectively. When statistical significance is present, the statistical significance marks have the color of the best endodontic motor.

All the results in reciprocating motion are reported in the Fig. 4 and Tables 6, 7.

**Table 6:** Statistically significant differences obtained in penetration during reciprocating tests (*P < 0.05; **P< 0.01; ***P < 0.001; ****P < 0.0001).

Continuous Rotation	Dual Move	Canal Pro CL2i	Jeni Motor	Ai Motor	Wave One motor	Smart A
Jeni Motor	15 mm** 16 mm** 16.5 mm* 17.5 mm*	11 mm** 14 mm*** 15 mm**** 16 mm**** 16.5 mm*** 17 mm*** 17.5 mm**** 18 mm***				
Ai motor	12.5 mm** 14 mm** 15 mm** 16 mm** 16.5 mm** 17 mm** 17.5 mm** 18 mm*	11 mm**** 12.5 mm**** 14 mm**** 15 mm**** 16 mm**** 16.5 mm**** 17 mm**** 17.5 mm**** 18 mm****				16.5 mm** 17 mm** 17.5 mm* 18 mm*
Wave One motor	12.5 mm* 14 mm* 15 mm* 16 mm** 16.5 mm** 17 mm* 17.5 mm* 18 mm***	11 mm**** 12.5 mm**** 14 mm**** 15 mm**** 16 mm**** 16.5 mm**** 17 mm**** 17.5 mm**** 18 mm****				16.5 mm* 17 mm** 18 mm*

**Table 7:** Statistically significant differences obtained in removal during reciprocating tests (*P < 0.05; **P < 0.01; ***P < 0.001; ****P < 0.0001).

Reciprocating Motion	Dual Move	Canal Pro CL2i	Jeni Motor	Ai Motor	Wave One motor	Smart A
Dual Move		11 mm**** 12.5 mm**** 14 mm** 15 mm**** 16 mm**** 16.5 mm**** 17 mm**** 17.5 mm**** 18 mm****		11 mm*		12.5 mm* 16 mm* 18 mm*
Jeni Motor		11 mm* 12.5 mm* 14 mm* 15 mm* 16 mm** 16.5 mm** 17.5 mm** 18 mm*				
Ai motor		12.5 mm* 14 mm**** 15 mm*** 16 mm* 16.5 mm** 17 mm** 17.5 mm* 18 mm*				
Wave One motor		11 mm** 12.5 mm**** 14 mm**** 15 mm**** 16 mm**** 16.5 mm**** 17 mm**** 17.5 mm**** 18 mm****				12.5 mm* 18 mm*
Smart A		11 mm** 17 mm*				

## Discussion

Concerning the reciprocating motion, Fidler showed that is a complex kinematic to apprehend, presenting more critical parameters than only angles and rotational speed [74]. He concluded that actual kinematics values differ from manufacturers’ declared values. In the same way, Irmak and Orhan (2017) showed that the actual kinematics of reciprocating endodontic motors differ from the manufacturers’ set values [73]. Braambati et al. studied the CCW angle, CW angle, speed at both directions, and standstill time at each change of directions and confirmed that none of the motors were able to reproduce faithfully the set movements [71]. Regarding the continuous rotation motion, Monteiro-Netto et al. studied the rotational speed parameters of three endodontic motors. They demonstrated that endodontic motors presented different behaviors between them [72].

In conclusion, there is a difference between the values recommended by the manufacturer and the values that the motors achieve. Taking this fact into consideration, our study looked at the potential clinical impact of using one motor more than another.

It is important to note that the One RECI instruments have the same cross-section as the One Curve instruments. The only difference is the One RECI step inversion allowing the practitioner to conventionally keep the main reciprocity angle in a counter-clockwise way. Therefore, the choice of these instruments was made to fix the geometry parameter, which has a very strong impact on the analysis of the penetration/removal forces.

In the first hand, endodontic motors obtain good results in terms of reproducibility during continuous rotation movements. The differences are most often of the order of 1 N, therefore considered not perceptible from a clinical point of view. Continuous rotation is limited to a rotational speed and a maximum torque setting. These easy-to-manage parameters could explain why all the endodontic motors obtain very close results to each other. However, we can note that the Jeni Motor obtains the best results in penetration and removal, followed by the Dual Move motor. This first conclusion underlines the interest of intelligent endodontic motors. Effectively, due to its specific assistance system that control the variable instrument movements, the Jeni Motor is the only tested motor in our work which can adapt the One Curve kinematics movements according to torsional stresses. This particularity is certainly the reason why the Jeni Motor achieves the best results during continuous rotation movements. The Canal Pro CL2i motor is the only motor that has reached the maximum torque limits during canal shaping, mainly in the medial and apical parts of the canals.

In the other hand, the results obtained during reciprocating motion are much difficult to analyze than those obtained during continuous rotation movements. Reciprocating settings are more complex and include (i) clockwise and anti-clockwise angle, (ii) overall speed of rotation, (iii) clockwise acceleration, (iv) counter-clockwise acceleration (v) maximum torque and (vi) standstill time during the direction changes. All motors are not programmable in the same way. For example, some motors do not present all the ranges of reciprocity angles, others do not allow to program the speed or the maximum torque in reciprocating mode. The differences inherent to each motor were important to consider and highlight the disparity in motor parameters. However, no contraindication to the use of endodontic instruments on these motors is mentioned, both on the instrument and motor instructions. In reciprocity, only the angles of rotation are systematically communicated. Therefore, a practitioner could use a motor with a non-recommended speed or torque due to the impossibility to program the motor. In our study, these parameters were deliberately not changed in order to reproduce clinical reality. Therefore, it is important to know the indications of each motor/instrument couple if some motor parameters are not programmable. At equivalent engine parameters, our results show very different penetration and withdrawal force values. In accordance with literature, this observation confirms that the declared manufacturers’ values are different from the really obtained ones [71–74].

The Jeni Motor angles are set by the combination of rotation speed and duration in milliseconds in clockwise/counter-clockwise directions. Therefore, the Jeni Motor is the only fully reciprocally programmable motor. Our study showed that the Jeni Motor reach one of the best results in reciprocating motion despite the lack of specific assistance for the One Reci. The Wave One motor is the oldest tested motor and is one of the first technologies on the market to have offered reciprocating motion. The good mastery of this movement could therefore explain its good results.

The Ai Motor obtained the best results for the 60°/170° angles, followed by the Jeni Motor. The Wave One motor obtained the best results for 30°/150° angles. In addition, it is interesting to note that the One RECI instruments (manufacturers recommendations 60 °CW/170 °CCW) showed better mechanical behavior at 30 °CW/150 °CCW with the Wave One Motor than at 60 °CW/170 °CCW with Dual Move, Ai Motor and Smart A motors.

The Ai Motor obtained excellent results in reciprocating motion, both during penetration and removal movements. The Dual Move also presented good results, mainly during removal movements. The Canal Pro CL2i obtained the worst results. This could be explained by a higher difference between the entered and obtained values of the Canal Pro CL2i motor than those of the other tested motors. Accordingly with this hypothesis, the root canal instruments were used in unfavorable conditions which could increase the stresses suffered.

## Conclusion

Within the limits of this study, we can highlight (i) that endodontic motors influence the mechanical behavior of endodontic instruments, (ii) that the influence of the motors is essentially objectified during reciprocating motion and (iii) that the reciprocating angles influence the mechanical behavior of endodontic instruments.

For continuous rotation and within the limits of our results, we obtained:

$${{{{{\rm{Jeni}}}}}}\; {{{{{\rm{Motor}}}}}} \, > \, {{{{{\rm{Dual}}}}}}\; {{{{{\rm{Move}}}}}} \, > \, [{{{{{\rm{Ai}}}}}}\; {{{{{\rm{Motor}}}}}}{-}{{{{{\rm{Wave}}}}}}\; {{{{{\rm{One}}}}}}{-}{{{{{\rm{Smart}}}}}}\; {{{{{\rm{A}}}}}}] \, > \, {{{{{\rm{CL}}}}}}2{{{{{\rm{i}}}}}}$$

For reciprocating motion results, taking account the correct or not correct parameter settings and within the limits of our results, we obtained:

$$\left[{{{{{\rm{Wave}}}}}}\; {{{{{\rm{One}}}}}}\; {{{{{\rm{Motor}}}}}}{-}{{{{{\rm{Ai}}}}}}\; {{{{{\rm{Motor}}}}}}{-}{{{{{\rm{Jeni}}}}}}\; {{{{{\rm{Motor}}}}}}\right] \, > \, {{{{{\rm{Dual}}}}}}\; {{{{{\rm{Move}}}}}} \, > \, {{{{{\rm{Smart}}}}}}\; {{{{{\rm{A}}}}}} \, > \, {{{{{\rm{Canal}}}}}}\; {{{{{\rm{Pro}}}}}}\; {{{{{\rm{CL}}}}}}2{{{{{\rm{i}}}}}}$$

## Data Availability

The datasets supporting the conclusions of this article are included within the article and its additional files.

## References

[1] Esposito PT, Cunningham CJ (1995). A comparison of canal preparation with nickel-titanium and stainless steel instruments. J Endod.

[2] Schrader C, Ackermann M, Barbakow F (1999). Step-by-step description of a rotary root canal preparation technique. Int Endod J.

[3] Bergmans L, Van Cleynenbreugel J, Wevers M, Lambrechts P (2001). Mechanical root canal preparation with NiTi rotary instruments: rationale, performance and safety. Status report for the American Journal of Dentistry. Am J Dent.

[4] Vincent M, Xolin P, Gevrey AM, Thiebaud F, Engels-Deutsch M, Ben Zineb T (2017). Experimental and numerical analysis of penetration/removal response of endodontic instrument made of single crystal Cu-based SMA: comparison with NiTi SMA instruments. Smart Mater Struct.

[5] Kabil E, Katić M, Anić I, Bago I (2021). Micro-computed evaluation of canal transportation and centering ability of 5 rotary and reciprocating systems with different metallurgical properties and surface treatments in curved root canals. J Endod.

[6] Sadeghi S (2011). Shaping ability of NiTi rotary versus stainless steel hand instruments in simulated curved canals. Med Oral. Patol Oral Cirugia Bucal.

[7] Akhlaghi NM, Dadresanfar B, Darmiani S, Moshari A (2014). Effect of master apical file size and taper on irrigation and cleaning of the apical third of curved canals. J Dent Tehran Iran.

[8] Capar ID, Arslan H, Akcay M, Uysal B (2014). Effects of ProTaper Universal, ProTaper Next, and HyFlex instruments on crack formation in dentin. J Endod.

[9] Kim HC, Kim HJ, Lee CJ, Kim BM, Park JK, Versluis A (2009). Mechanical response of nickel-titanium instruments with different cross-sectional designs during shaping of simulated curved canals. Int Endod J.

[10] Schäfer E, Tepel J (2001). Relationship between design features of endodontic instruments and their properties. Part 3. Resistance to bending and fracture. J Endod.

[11] Dablanca-Blanco AB, Castelo-Baz P, Miguéns-Vila R, Álvarez-Novoa P, Martín-Biedma B (2022). Endodontic rotary files, what should an endodontist know?. Med Kaunas Lith.

[12] Ruddle CJ (2000). Nickel-titanium rotary systems: review of existing instruments and geometries. Dent Today.

[13] Ferraz CC, Gomes NV, Gomes BP, Zaia AA, Teixeira FB, Souza-Filho FJ (2001). Apical extrusion of debris and irrigants using two hand and three engine-driven instrumentation techniques. Int Endod J.

[14] Yared G (2008). Canal preparation using only one Ni-Ti rotary instrument: preliminary observations. Int Endod J.

[15] Schäfer E, Schulz-Bongert U, Tulus G (2004). Comparison of hand stainless steel and nickel titanium rotary instrumentation: a clinical study. J Endod.

[16] Ahmad MZ, Sadaf D, MacBain MM, Merdad KA (2022). Effect of mode of rotation on apical extrusion of debris with four different single-file endodontic instrumentation systems: Systematic review and meta-analysis. Aust Endod J J Aust Soc Endodontol Inc.

[17] Pedullà E, Grande NM, Plotino G, Gambarini G, Rapisarda E (2013). Influence of continuous or reciprocating motion on cyclic fatigue resistance of 4 different nickel-titanium rotary instruments. J Endod.

[18] Orhan EO, Bahadır D, Irmak O (2022). Kinematics of « Adaptive Motion » under constant torque values. J Endod.

[19] Ferreira F, Adeodato C, Barbosa I, Aboud L, Scelza P, Zaccaro Scelza M (2017). Movement kinematics and cyclic fatigue of NiTi rotary instruments: a systematic review. Int Endod J.

[20] Ahn SY, Kim HC, Kim E (2016). Kinematic effects of Nickel-Titanium instruments with reciprocating or continuous rotation motion: a systematic review of in vitro studies. J Endod.

[21] Çapar ID, Arslan H (2016). A review of instrumentation kinematics of engine-driven nickel-titanium instruments. Int Endod J.

[22] Pedrinha VF, Brandão JMdaS, Pessoa OF, Rodrigues PdeA (2018). Influence of file motion on shaping, apical debris extrusion and dentinal defects: a critical review. Open Dent J.

[23] Anderson ME, Price JWH, Parashos P (2007). Fracture resistance of electropolished rotary nickel-titanium endodontic instruments. J Endod.

[24] Rapisarda E, Bonaccorso A, Tripi TR, Fragalk I, Condorelli GG (2000). The effect of surface treatments of nickel-titanium files on wear and cutting efficiency. Oral Surg Oral Med Oral Pathol Oral Radiol Endod.

[25] Boessler C, Paque F, Peters OA (2009). The effect of electropolishing on torque and force during simulated root canal preparation with ProTaper shaping files. J Endod.

[26] Mohammadi Z, Soltani MK, Shalavi S, Asgary S (2014). A review of the various surface treatments of NiTi instruments. Iran. Endod. J.

[27] Gavini G, Pessoa OF, Barletta FB, Vasconcellos MaZ, Caldeira CL (2010). Cyclic fatigue resistance of rotary nickel-titanium instruments submitted to nitrogen ion implantation. J Endod.

[28] Ebihara A, Yahata Y, Miyara K, Nakano K, Hayashi Y, Suda H (2011). Heat treatment of nickel-titanium rotary endodontic instruments: effects on bending properties and shaping abilities. Int Endod J.

[29] Goo HJ, Kwak SW, Ha JH, Pedullà E, Kim HC (2017). Mechanical properties of various heat-treated nickel-titanium rotary instruments. J Endod.

[30] Hou XM, Yang YJ, Qian J (2020). Phase transformation behaviors and mechanical properties of NiTi endodontic files after gold heat treatment and blue heat treatment. J Oral Sci.

[31] Miyara K, Yahata Y, Hayashi Y (2014). The influence of heat treatment on the mechanical properties of Ni-Ti file materials. Dent Mater J.

[32] Zupanc J, Vahdat-Pajouh N, Schäfer E (2018). New thermomechanically treated NiTi alloys - a review. Int Endod J.

[33] Shen Y, Zhou Hmin, Zheng Yfeng, Peng B, Haapasalo M (2013). Current challenges and concepts of the thermomechanical treatment of nickel-titanium instruments. J Endod.

[34] Testarelli L, Plotino G, Al-Sudani D (2011). Bending properties of a new nickel-titanium alloy with a lower percent by weight of nickel. J Endod.

[35] Schäfer E, Dzepina A, Danesh G (2003). Bending properties of rotary nickel-titanium instruments. Oral Surg Oral Med Oral Pathol Oral Radiol Endod.

[36] Özyürek T, Gündoğar M, Yılmaz K, Uslu G (2017). Bending resistance and cyclic fatigue life of Reciproc Blue, WaveOne Gold, and Genius files in a double (S-shaped) curved canal. J Dent Res Dent Clin Dent Prospects.

[37] Oh S, Kum KY, Kim HJ (2020). Bending resistance and cyclic fatigue resistance of WaveOne Gold, Reciproc Blue, and HyFlex EDM instruments. J Dent Sci..

[38] De-Deus G, Silva EJNL, Vieira VTL (2017). Blue thermomechanical treatment optimizes fatigue resistance and flexibility of the reciproc files. J Endod.

[39] Yahata Y, Yoneyama T, Hayashi Y (2009). Effect of heat treatment on transformation temperatures and bending properties of nickel-titanium endodontic instruments. Int Endod J.

[40] Hamdy TM, Galal M, Ismail AG, Abdelraouf RM (2019). Evaluation of flexibility, microstructure and elemental analysis of some contemporary nickel-titanium rotary instruments. Open Access Maced. J. Med Sci.

[41] Abdelmomen I, Vincent M, Thiebaud F (2023). Experimental analysis of the influence of heat treatments on the flexibility of NiTi alloy for endodontic instruments manufacturing. Materials.

[42] Hieawy A, Haapasalo M, Zhou H, Wang ZJ, Shen Y (2015). Phase transformation behavior and resistance to bending and cyclic fatigue of ProTaper Gold and ProTaper Universal Instruments. J Endod.

[43] Oh S, Kum KY, Cho K (2019). Torsional and bending properties of V Taper 2H, ProTaper NEXT, NRT, and one shape. BioMed. Res Int.

[44] Berutti E, Chiandussi G, Gaviglio I, Ibba A (2003). Comparative analysis of torsional and bending stresses in two mathematical models of nickel-titanium rotary instruments: ProTaper versus ProFile. J Endod.

[45] Xu X, Eng M, Zheng Y, Eng D (2006). Comparative study of torsional and bending properties for six models of nickel-titanium root canal instruments with different cross-sections. J Endod.

[46] Rubio J, Zarzosa JI, Aranda S, Casino A, Pallarés A (2022). A comparative study of cyclic fatigue of 6 endodontic systems. An in vitro study. J Clin Exp Dent.

[47] Larsen CM, Watanabe I, Glickman GN, He J (2009). Cyclic fatigue analysis of a new generation of nickel titanium rotary instruments. J Endod.

[48] Vadhana S, SaravanaKarthikeyan B, Nandini S, Velmurugan N (2014). Cyclic fatigue resistance of RaCe and Mtwo rotary files in continuous rotation and reciprocating motion. J Endod.

[49] Tripi TR, Bonaccorso A, Condorelli GG (2006). Cyclic fatigue of different nickel-titanium endodontic rotary instruments. Oral Surg Oral Med Oral Pathol Oral Radiol Endod.

[50] Pruett JP, Clement DJ, Carnes DL (1997). Cyclic fatigue testing of nickel-titanium endodontic instruments. J Endod f.évr.

[51] Bahia MGA, Buono VTL (2005). Decrease in the fatigue resistance of nickel-titanium rotary instruments after clinical use in curved root canals. Oral Surg Oral Med Oral Pathol Oral Radiol Endod.

[52] Haïkel Y, Serfaty R, Bateman G, Senger B, Allemann C (1999). Dynamic and cyclic fatigue of engine-driven rotary nickel-titanium endodontic instruments. J Endod.

[53] Ullmann CJ, Peters OA (2005). Effect of cyclic fatigue on static fracture loads in ProTaper nickel-titanium rotary instruments. J Endod.

[54] Kuhn G, Jordan L (2002). Fatigue and mechanical properties of nickel-titanium endodontic instruments. J Endod.

[55] Chaves Craveiro de Melo M, Guiomar de Azevedo Bahia M, Lopes Buono VT (2002). Fatigue resistance of engine-driven rotary nickel-titanium endodontic instruments. J Endod.

[56] Kiefner P, Ban M, De-Deus G (2014). Is the reciprocating movement per se able to improve the cyclic fatigue resistance of instruments?. Int Endod J.

[57] Campos GO, Fontana CE, Vieira VTL, Elias CN, de Martin AS, Bueno CEdaS. Influence of heat treatment of nickel-titanium instruments on cyclic fatigue resistance in simulated curved canals. Eur J Dent. 2023;17:472–7. 10.1055/s-0042-1747952 PMC10329553 36195211

[58] Yared G, Kulkarni GK, Ghossayn F (2003). An in vitro study of the torsional properties of new and used K3 instruments. Int Endod J.

[59] Pereira ESJ, Singh R, Arias A, Peters OA (2013). In vitro assessment of torque and force generated by novel ProTaper Next Instruments during simulated canal preparation. J Endod.

[60] Gomes MS, Vieira RM, Böttcher DE, Plotino G, Celeste RK, Rossi-Fedele G (2021). Clinical fracture incidence of rotary and reciprocating NiTi files: A systematic review and meta-regression. Aust Endod J Aust Soc Endodontol Inc.

[61] Sattapan B, Nervo GJ, Palamara JE, Messer HH (2000). Defects in rotary nickel-titanium files after clinical use. J Endod.

[62] Abou El Nasr HM, Abd El Kader KG (2014). Dentinal damage and fracture resistance of oval roots prepared with single-file systems using different kinematics. J Endod.

[63] Parashos P, Gordon I, Messer HH (2004). Factors influencing defects of rotary nickel-titanium endodontic instruments after clinical use. J Endod.

[64] Krikeli E, Mikrogeorgis G, Lyroudia K (2018). In vitro comparative study of the influence of instrument taper on the fracture resistance of endodontically treated teeth: an integrative approach-based analysis. J Endod.

[65] Spili P, Parashos P, Messer HH (2005). The impact of instrument fracture on outcome of endodontic treatment. J Endod.

[66] Altenburger MJ, Cenik Y, Schirrmeister JF, Wrbas KT, Hellwig E (2009). Combination of apex locator and endodontic motor for continuous length control during root canal treatment. Int Endod J.

[67] Christofzik DW, Bartols A, Khaled M, Größner-Schreiber B, Dörfer CE (2017). The accuracy of the auto-stop function of different endodontic devices in detecting the apical constriction. BMC Oral Health.

[68] Gambarini G, Piasecki L, Miccoli G (2019). Classification and cyclic fatigue evaluation of new kinematics for endodontic instruments. Aust Endod J J Aust Soc Endodontol Inc.

[69] Gambarini G (2001). Cyclic fatigue of nickel-titanium rotary instruments after clinical use with low- and high-torque endodontic motors. J Endod.

[70] Iacono F, Pirani C, Arias A (2019). Impact of a modified motion on the fatigue life of NiTi reciprocating instruments: a Weibull analysis. Clin Oral Investig.

[71] Braambati D, Monteiro Netto RdeC, Coelho MS, Soares AdeJ, Frozoni M (2022). Reciprocating Kinematics of X-Smart Plus, VDW Silver and, iRoot endodontic motors: a comparison between real and set values. Braz Dent J.

[72] Monteiro-Netto RdeC, Braambati D, Arruda-Vasconcelos R, Soares Ade-Jesus, Frozoni M (2023). Evaluation of the rotary kinematics between actual and set speeds of X-Smart Plus, VDW.Silver and iRoot motors. Braz Dent J.

[73] Irmak Ö, Orhan EO. Kinematic analysis of new and used reciprocating endodontic motors in 2 different modes. Int J Artif Organs. 2017;0. 10.5301/ijao.5000640. 10.5301/ijao.5000640 28862719

[74] Fidler A (2014). Kinematics of 2 reciprocating endodontic motors: the difference between actual and set values. J Endod.

[75] Bürklein S, Stüber JP, Schäfer E (2019). Real-time dynamic torque values and axial forces during preparation of straight root canals using three different endodontic motors and hand preparation. Int Endod J.

[76] Gambarini G (2000). Rationale for the use of low-torque endodontic motors in root canal instrumentation. Endod Dent Traumatol.

[77] Yared G, Bou Dagher F, Kulkarni K (2003). Influence of torque control motors and the operator’s proficiency on ProTaper failures. Oral Surg Oral Med Oral Pathol Oral Radio Endod.

[78] Zarei M, Javidi M, Erfanian M, Lomee M, Afkhami F (2013). Comparison of air-driven vs electric torque control motors on canal centering ability by ProTaper NiTi rotary instruments. J Contemp Dent Pr.

[79] Cruz ATG, Wichnieski C, Carneiro E, da Silva Neto UX, Gambarini G, Piasecki L (2017). Accuracy of 2 Endodontic rotary motors with integrated apex locator. J Endod.

[80] Maree-Ali M, Wigler R, Lin S, Kaufman AY (2016). Erratum to: An ex vivo comparison of working length determination by three electronic root canal length measurement devices integrated into endodontic rotary motors. Clin Oral Investig.

[81] Bernardes RA, Feitosa APOP, Bramante CM (2022). Evaluation of foramen locating accuracy of an endodontic motor integrated with electronic foramen employing optimal glide path kinematics. Clin Oral Investig.

[82] Uzun O, Topuz O, Tinaz C, Nekoofar MH, Dummer PMH (2008). Accuracy of two root canal length measurement devices integrated into rotary endodontic motors when removing gutta-percha from root-filled teeth. Int Endod J.

[83] Paiva HC, Akisue E, de Miranda Candeiro GTC, de Lima Scardini I, Caldeira CL, Gavini G (2022). Influence of heat treatment of nickel-titanium instruments on the accuracy of an electronic apex locator integrated with endodontic motor. J Conserv Dent.

[84] Koçak S, Koçak MM, Sağlam BC (2013). Efficiency of 2 electronic apex locators on working length determination: A clinical study. J Conserv Dent.

[85] Vincent M. Développement d’un instrument endodontique en alliage à mémoire de forme monocristallin cuivreux. Thesis PhD, 2017.

